# Oncoplastic Surgery in Japanese Patients with Breast Cancer Close to the Areola: Partial Mastectomy Using Periareolar Mammoplasty: A Case Report

**DOI:** 10.1155/2011/121985

**Published:** 2011-09-11

**Authors:** Yuko Kijima, Heiji Yoshinaka, Munetsugu Hirata, Tadao Mizoguchi, Sumiya Ishigami, Akihiro Nakajo, Hideo Arima, Shinichi Ueno, Shoji Natsugoe

**Affiliations:** Department of Digestive Surgery, Breast and Thyroid Surgery, Kagoshima University Graduate School of Medical and Dental Sciences, 8-35-1 Sakuragaoka, Kagoshima 890-8520, Japan

## Abstract

We report the results of oncoplastic surgery in two Japanese patients with early breast cancer. Their breasts were large and ptotic, and their lesions, which were close to the areola, were considered to be suitable for breast conservative surgery. Oncoplastic surgery involving partial resection of the gland and a periareolar mammoplasty were performed. The technique was easy to perform, and the cosmetic outcome was excellent.

## 1. Introduction

Oncoplastic techniques have succeeded in expanding the role of breast conserving therapy (BCT) to early breast cancer lesions [1]. There are many different oncoplastic surgical techniques, one of which involves careful planning of skin and parenchymal excisions, reshaping of the gland after the parenchymal excision, and repositioning of the NAC to the center of the breast mound with or without correction of the contralateral breast for better symmetry [2]. It is reported that oncoplastic surgery involving partial resection of the gland and periareolar mammoplasty yields very satisfactory cosmetic results and minimal complications, and it is considered to be an appropriate therapeutic option for patients with lesions close to the areola [3, 4]. We performed this procedure on a Japanese patient with large and ptotic breasts.

## 2. Case Report

A 51-year-old Japanese lady was referred to our institution due to a cystic lesion in the upper-inner quadrant of her left breast close to the NAC, which was detected by computed tomography (CT) performed as a preoperative systemic study for carcinoma uteri. On CT, the breast lesion was suspected to be an intracystic tumor. The patient was premenopausal and had no history of pregnancy. A preoperative study using mammography, ultrasonography, CT, histological examinations (fine needle aspiration biopsy and core needle biopsy), bone scintigraphy, and magnetic resonance imaging was performed. The lesion was found to have a solid component but no cytological or histological diagnosis was obtained. She underwent hysterectomy under general anesthesia at our hospital, during which we performed excisional biopsy of her left breast lesion and sentinel lymph node biopsy. After histological examination of the permanent sample, she was diagnosed with noninvasive ductal carcinoma with positive surgical margins and an intraductal component although no sentinel lymph nodes metastasized. She did not suffer from any systemic diseases such as diabetes mellitus and did not smoke. Her breasts were relatively large and ptotic, and her NAC was located beneath the inframammary line. We therefore decided to perform oncoplastic surgery combining with partial mastectomy and recentralization of NAC in order to achieve appropriate oncological and cosmetic outcomes, and surgical approach for contralateral healthy breast for better symmetrical results. Informed consent was obtained from her prior to the oncoplastic surgery.

## 3. Design

**Figure 1:**
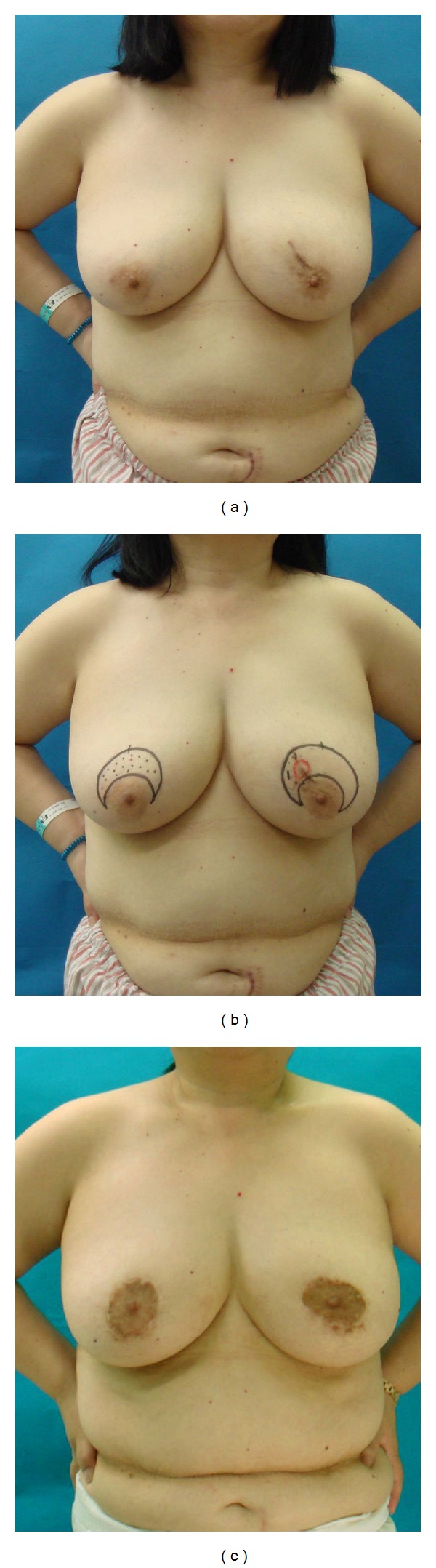
*Case  1*. A 51-year-old patient with a T1 tumor in the upper-inner area of her left breast. (a) Bilateral ptotic breasts with the nipple-areola complex (NAC) located beneath the inframammary line. The surgical scar produced by the excisional biopsy was located at the 11 O'clock position of her left breast. (b) Cystic degeneration caused by the excision biopsy was detected by ultrasonography. Periareolar mammaplasty was planned. (c) Postoperative 18 months.

On the day before surgery, the patient was seen by the breast surgeon so that he could plan the operation, make drawings, and explain the different surgical options. The excisional biopsy had left a 3 cm radial scar at the 11 o'clock position of her left breast (Figure 1(a)). With the patient in a standing position, the surgeon made sure that the nipple was located below the inframammary line. With the patient in a supine position, the area affected by cystic degeneration caused by the excisional biopsy, which was 1.0 cm in diameter, was determined by ultrasonographic examination and marked on the skin surface. Then, the crescent-shaped partial mastectomy line was marked on her skin using permanent ink (Figure 1(b)).

## 4. Surgical Procedure

**Figure 2:**
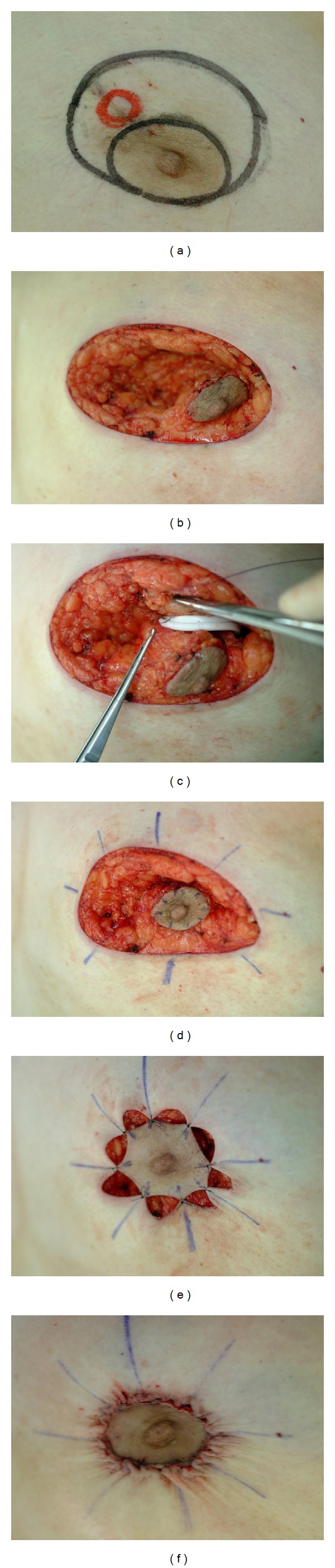
Preoperative design of the periareolar technique showing the tumor. (a) A crescent of skin was removed together with a partial gland. A residual cancer lesion was suspected (red circle). (b) A cylinder of gland tissue was removed together with the pectoralis major muscle. (c) A suction tube was left on the surface of pectoral major muscle. (d) The superior and inferior pedicles were sutured to reduce the defect. (e) The areola suturing was completed with a single suture 4-0 PDS and a running subcuticular 4-0 Monocryl. (f) After closure.

**Figure 3:**
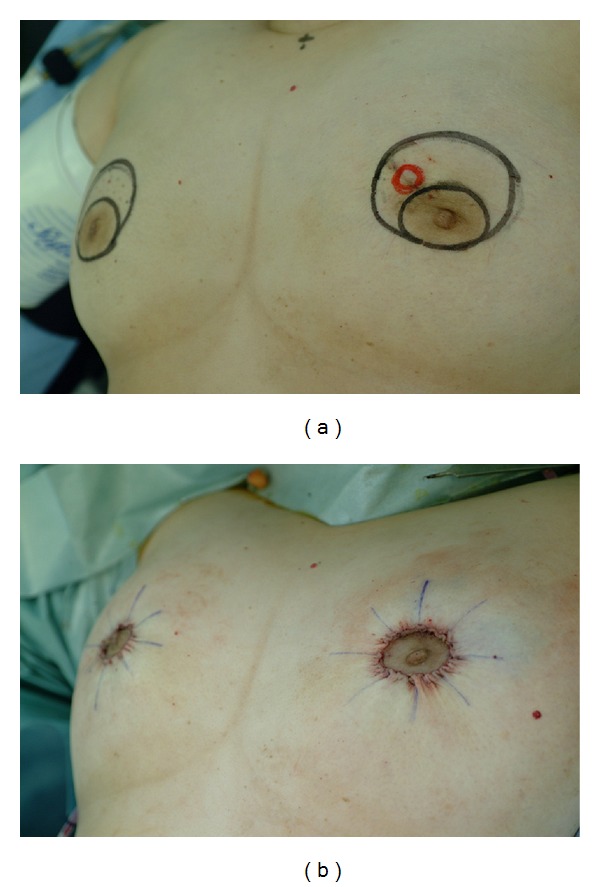
Pre- and postoperative images of case 1. (a) A crescent of skin and the parenchymal tissue just under it were removed. (b) Identical bilateral procedures were performed.

**Figure 4:**
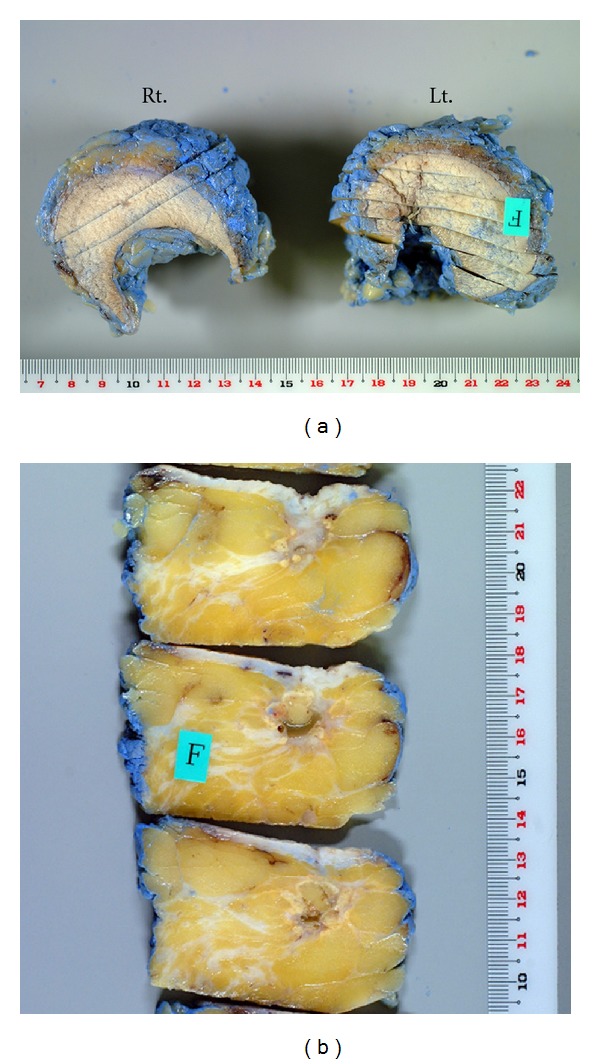
Bilateral resected tissue. (a) The resected tissues weighed 94 g (right) and 104 g (left), respectively. (b) Fixed and sliced tissue used for the histological examination. No residual cancer cells were seen in the left breast.

The surgical procedure began with partial mastectomy including sufficient surgical margins and a full-thickness glandular excision (Figure 2(a)). The fascia of the pectoral major muscle just below the resected area was completely removed, as determined by the preoperative drawings (Figure 2(b)). During the operation, several surgical margins containing tissue underlying the NAC were histologically examined to ensure that the cancerous lesion had been completely removed. No marginal invasion was detected during the intraoperative examination and so the superior and inferior pedicles were sutured (Figures 2(c) and 2(d)). A contralateral procedure was also added. Mirror image biopsy was selected, and partial mastectomy was performed in the same manner as for the treated breast (Figure 3(a)), and 94 g and 104 g tissue were resected in the contralateral and treated breast, respectively (Figure 4(a)). Closed suction drainage lines were placed onto the surfaces of the bilateral pectoralis major muscles. The bilateral NAC were located at medial-upper positions on the breast mounds (Figures 2(e), 2(f), and 3(b)). 

In our institution, postoperative radiotherapy is administered to selected patients whose lesions are located within 10 mm of the surgical margin and contain both invasive and intraductal components. The present case did not require radiotherapy as no remnant cancer lesion was observed in the additional resected tissue (Figure 4(b)). There has been no distant or local recurrence during eighteen months postoperative followup, and excellent symmetry was obtained.

**Figure 5:**
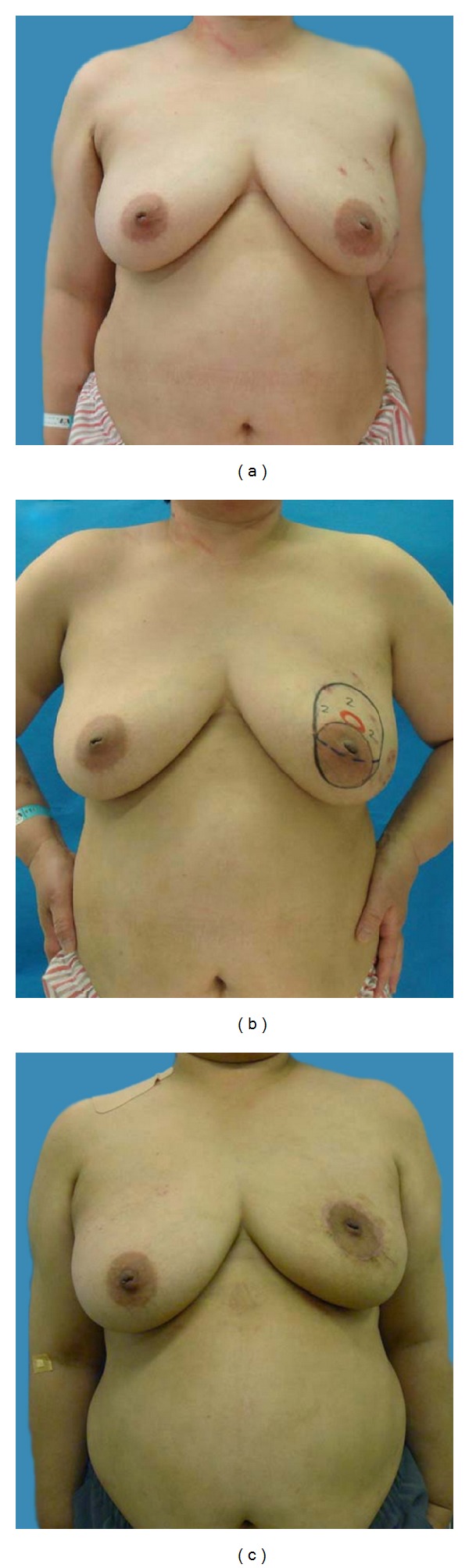
*Case  2*. a 51-year-old patient with a T1 tumor in the periareolar area of her left breast. (a) Her breasts were ptotic and large. (b) Lesions were detected by ultrasonography with the patient in a supine position. A 2 cm surgical margin (black circle) was drawn around the cancer lesion (red circle). No contralateral procedure was planned. (c) Postoperative 6 months.

 A 51-year-old lady with large ptotic breasts was diagnosed with a T1 tumor located at the 1 O'clock position of her left breast (Figure 5). According to her wishes, no contralateral procedure was added. As a result, the heights of the lowest points of her bilateral breasts and nipples were different, but no breast deformity occurred in the treated breast. In cases 1 and 2, the total operative duration was 155 and 121 minutes and the duration of the plastic surgery was 119 and 66 minutes, respectively.

## 5. Discussion

Oncoplastic techniques, which combine the concepts of oncologic and plastic surgery, are becoming more common, especially in Western countries [1, 5]. There are many different oncoplastic techniques, one of which involves careful planning of skin and parenchymal excisions, reshaping of the gland after the parenchymal excision, and repositioning of the NAC to the center of the breast mound with or without correction of the contralateral breast for better symmetry [6]. Oncoplastic surgical procedures have been discussed in many reports. The existing mammoplasty techniques were initially adapted to produce oncoplastic surgical procedures for specific tumor locations such as cancers involving the periareolar area [2, 7, 8]. However, in Japan, the use of such techniques combining partial mastectomy and the reduction type of surgery is rare, although a few case reports in which oncoplastic surgery involving a combination of reduction mammoplasty and NAC recentralization was performed for Japanese patients with breast cancer have been published [9, 10]. 

We have introduced an oncoplastic surgery combining partial mastectomy and immediate volume replacement from autologous extramammary tissue [11, 12]. For patients with cancer lesions in medial or central areas, we have been able to easily repair the defect using a distant free dermal fat graft [13, 14]. On the other hand, for patients with relatively large and ptotic breasts, oncoplastic surgery combining a reduction type operation and recentralization of the NAC produced good results [9, 15]. In another patient with ptotic breasts, who was diagnosed with ductal carcinoma in situ in the lower area of the breast with intraductal spread to the nipple, we successfully performed an oncoplastic procedure involving an amputation-type partial mastectomy and grafting of the NAC [16]. From these experiences, we now indicate reduction type oncoplastic surgery rather than volume replacement procedures for patients with large or ptotic breasts, such as Western women [17]. 

Berry et al. reviewed cases treated with oncoplastic surgery and classified them according to tumor location [4]. In their review, they recommended that a periareolar approach is ideal for tumors close to the areolar (mostly upper pole tumors) in mildly ptotic breasts that would benefit from mastopexy based on Benelli's “round block technique” [18]. According to previous reports on breast periareolar aesthetic surgery, the main advantage of breast periareolar aesthetic surgery is the small residual scar produced around the areola, which is generally less conspicuous than the scars produced by conventional techniques, and the main disadvantage is that this technique cannot be used to correct ptosis or flabbiness of the breast [19–23]. 

Avoiding NAC displacement is a key aim of all oncoplastic surgery. The NAC is repositioned to adjust for both the anticipated deviation and the new shape of the breast. We achieved an excellent cosmetic outcome in case 1, who received bilateral oncoplastic surgery whereas an asymmetric outcome was seen in case 2, who underwent unilateral surgery. However, no nipple deviation toward the area of excision due to fibrosis-induced tension after partial mastectomy occurred in either patient. 

Mammoplasty techniques for cosmetic breast reduction have a low complication rate of 1-2%. Early common complications include seroma, hematoma, infection, and skin or NAC necrosis leading to delayed healing. Late complications may involve fat necrosis, loss of nipple sensitivity, and necrosis [24, 25]. Fitoussi et al. [3] reported the results of oncoplastic surgery for central tumors, including the complications and histological findings. Out of 146 patients, 14% underwent periareolar techniques. Symmetrising surgery was performed simultaneously in 17% of cases, and the complication rate was 9% (the most common complications were hematomas, wound breakdown, delayed healing, and infection) [2]. Clear excision margins were maintained in more than 80% of patients. The remaining patients with incomplete margins were treated with completion mastectomy (9 patients), a further wide excision plus radiotherapy (2 patients), or an additional boost of local radiotherapy (12 patients). Secondary NAC reconstruction was performed in a third of the patients. In the patients who underwent oncoplastic surgery involving the periareolar area, after examination of the surgical margins, it was concluded that adequate oncological outcomes had been achieved; that is, equivalent to that achieved in the patients who underwent breast-conserving surgery without any plastic procedure. 

Although only two cases were reported in this paper and the follow-up period was short, we have revealed that oncoplastic surgery for patients with cancer lesions close to the nipple is oncologically safe and produced excellent outcomes especially in the patient who received bilateral mammaplasty. It is a relatively simple procedure that yields very satisfactory cosmetic results with minimal complications, and it may be considered a suitable therapeutic option for Japanese women with large breasts as well as Western women.

## 6. Conclusion

This result indicates that oncoplastic surgery involving a periareolar approach can be used for BCT in Japanese patients with cancer lesions close to the NAC of large breasts and achieves an excellent outcome and oncological control.
